# Atlanta Academy of Medicine

**Published:** 1878-05

**Authors:** J. G. Westmoreland

**Affiliations:** Reporter


### Reports of Societies.

### ATLANTA ACADEMY OF MEDICINE.

J. G. WESTMORELAND, M.D., Reporter.

Atlanta, Ga., April 1, 1878.

Vice-President J. T. Johnson in the chair.

Dr. Calhoun reported the case of a man fifty years old having hypertrophy of the epiglottis. This part has a granular appearance, with excessive secretion of muco-pus. The aretinoid cartilages also partake of the diseased condition, leaving, however, the vocal cords nearly or quite natural. The history gives no reason to suspect cancerous or syphilitic taint. Dr. C. suspects malignant growth, however, and would be pleased to have the opinion of members.

Dr. J. G. Westmoreland reported a case of injury to a man 60 years old, by being precipitated from a bridge some ten feet high, with a horse and buggy. The accident occurred on Monday, the 25th of March, and he saw him first on Wednesday the 27th. At first the most prominent difficulty seemed to exist in the urethra. A catheter had been introduced and left remaining, but imperfectly conveyed the urine from the bladder. As reported by a physician present at the time of injury, hemorrhage occurred from the urethra soon after the fall, and retention of urine existed until a catheter was introduced. At first the instrument seemed to pass a sufficient distance to reach the bladder without discharging any urine, and only by manipulation with a finger in the rectum so as to press the end of the catheter firmly against the anterior wall of the urethra, could it be made to enter the bladder. This fact, with that of urethral hemorrhage soon after the injury, led to the opinion of lacerated urethra. Evidently a severe blow had been received on the perineum, as the external surface of this part was very dark from the bruise. The catheter was withdrawn sometime during the day on Wednesday, and the urine passed sufficiently free not to require the re-introduction. In addition to the urethral trouble, there seemed to have been serious injury done to the right side of the abdomen and hip. The patient could not be moved in bed without great suffering in these parts. He had been the subject of inguinal hernia of the right side for several years, and was wearing a truss at the time of injury, in the course of which from the groin to the back, the skin was black from ecchymosis, showing that a severe blow had been received upon the truss. Enema of warm water caused the discharge of fæces contained in the lower bowels, but no more evacuation could be produced by cathartics and injections of large quantities of water into the bowels repeatedly. Repeated vomiting of dark fluid occurred during Wednesday and Thursday, and the circulation had increased from one hundred to a hundred and twenty in these two days. Though the excessive tenderness usually found in peritonitis was not present, yet there was sufficient evidence of extensive peritoneal inflammation. On Thursday night opium, which had been used moderately before, was given to almost complete narcotism, with the effect of relieving the vomiting entirely and reducing the pulse to about one hundred beats per minute. This condition remained until Saturday morning, when, with cool, damp surface, the circulation commenced declining in force, and gradually the vital powers gave way until death ensued on Monday about noon. The points of interest in this case are: the nature and location of the injury to the bowels, obstructing the passage of contents of the small intestines, and the cause of the excessive tympanitis which existed from the second day after the injury. Is it not probable that the cœcum, including the illeo-cœcal connection, was in the inguinal canal at the time the truss was forcibly driven against it, and thus so bruised and injured that no passage of fæces could occur? At first a suspicion of laceration of the bowel was entertained, but had been discarded, and that of injury to the illeo-cœcal valve adopted. A *post mortem* will probably be obtained.

---

ATLANTA, GA., April 8, 1878.

The President, Dr. J. F. Alexander, in the chair.

Dr. J. T. Johnson reported a case which he had seen in connection with Dr. Pinckney. The case was a man 52 years old, having typhoid symptoms, with alternation of constipation and diarrhœa. A tumor in the right illiac fossa, was discovered, and, proving a permanent and increasing enlargement, its contents were tested with the aspirator and found to be offensive pus, of which a considerable quantity was dischargad, by an incision with bistoury, greatly to the relief of the patient. He has improved decidedly since the evacuation, and hopes are entertained of his recovery. The abscess was supposed to be connected with the cæcum or appendix.

Dr. J. G. Westmoreland reported the result of autopsy in the case of fatal injury reported at last meeting, as follows:

A circular cut was made just above the pubis, so as to raise the abdominal wall in front, exposing the viscera completely. The whole peritoneum lining the cavity in front was extremely dark from congestion and inflammation; also that covering the right illiac fossa and psoas muscles. The entire cæcum, three or four inches of the ascending colon, and several inches of the illium exhibited evidences of a high degree of inflammation. The same dark appearance was found in the mesentery connected with the small intestines their whole extent, and in various places surrounding the bowel, for several inches in extent. Evidently the cæcum, colon and illium, at their junction, constituted the most prominent point of injury, and to this, as was expected, is certainly due the obstruction to the passage of contents of small intestines through the colon. Whether the cæcum was in the inguinal canal, as was supposed, and injured by a blow received upon the truss, or not, of course could not be certainly ascertained, but from the external discoloration being of the shape and in the position of the truss, such would seem to be the most reasonable conclusion. The bladder was found pretty firmly contracted, and showed less signs of inflammation than the peritoneum above.

Dr. Baird, in answer to an inquiry from the President, said he had only had an opportunity of testing the value of vaccinia as a cure for whooping cough in one case. A girl aged four years, the child of intelligent and cultivated parents, had suffered for two weeks from the disease. The paroxysms were frequent and violent. He vaccinated her upon the arm. The vesicles were beautifully developed, and after the fifth day the cough lost its characteristic whoop, the paroxysms became less and less frequent and severe, and long before the formation of the crusts, the child—with the exception of an occasional slight cough in the evening—appeared perfectly well. He was not prepared to say that the vaccination had effected a cure though the parents of the child expressed the greatest satisfaction with the results and are firm believers in its efficacy. One case proves nothing, but taken in connection with others is of value, in enabling us to arrive at a definite conclusion in regard to this question. He spoke favorably of the inhalation of carbolic acid as a remedy in whooping cough. In a case recently treated, after the failure of several remedies to alleviate the violence of the cough, marked improvement followed the exhibition of a combination of hydrate of chloral, bromide of potassium, hydrocyanic acid, ipecac. and opium.

